# Patshitinikutau Natukunisha Tshishennuat Uitshuau (a place for Elders to spend their last days in life): a qualitative study about Innu perspectives on end-of-life care

**DOI:** 10.1186/s12904-024-01431-5

**Published:** 2024-05-17

**Authors:** Russell Dawe, Jack Penashue, Mary Pia Benuen, Anastasia Qupee, Andrea Pike, Melanie van Soeren, Carolyn Sturge Sparkes, Mercy Winsor, Kristin Harris Walsh, Hiliary Hasan, Nathaniel Pollock

**Affiliations:** 1 Memorial University, St. John’s, Canada; 2 Innu Nation, Sheshatshiu, Canada; 3 Sheshatshiu Innu First Nation, Sheshatshiu, Canada; 4 School of Arctic and Subarctic Studies, Labrador Campus, Memorial University, Happy Valley-Goose Bay, Canada

**Keywords:** Innu, Sheshatshiu, Palliative care, End-of-life care, Indigenous, Patient-oriented research, Innu-aimun

## Abstract

**Background:**

Indigenous palliative persons and their families often have different values, spiritual traditions, and practices from Western culture and Canadian health systems. Additionally, many healthcare policies and practices have been established without adequate consultation of the Indigenous populations they are meant to serve. This can result in barriers to Innu receiving culturally safe end-of-life care. Innu community leaders from Sheshatshiu, Labrador, have identified a need for further research in this area. The purpose of this study is to: (1) describe the cultural and spiritual practices related to death and dying of the Innu in Sheshatshiu; (2) identify aspects of current end-of-life care delivery that serve and/or fail to meet the cultural and spiritual needs of the Innu in Sheshatshiu; and (3) explore ways to integrate current end-of-life care delivery practices with Innu cultural and spiritual practices to achieve culturally safer care delivery for the Innu.

**Methods:**

This qualitative patient-oriented research study was co-led by Innu investigators and an Innu advisory committee to conduct semi-structured interviews of 5 healthcare providers and 6 decision-makers serving the community of Sheshatshiu and a focus group of 5 Innu Elders in Sheshatshiu. Data was analyzed thematically from verbatim transcripts. The codebook, preliminary themes, and final themes were all reviewed by Innu community members, and any further input from them was then incorporated. Quotations in this article are attributed to Innu Elders by name at the Elders’ request.

**Results:**

The findings are described using eight themes, which describe the following: relationships and visitation support a “peaceful death”; traditional locations of death and dying; the important role of friends and community in providing care; flexibility and communication regarding cultural practices; adequate and appropriate supports and services; culturally-informed policies and leadership; and Innu care providers and patient navigators.

**Conclusions:**

The Innu in Sheshatshiu have a rich culture that contributes to the health, care, and overall well-being of Innu people approaching end of life. Western medicine is often beneficial in the care that it provides; however, it becomes culturally unsafe when it fails to take Innu cultural and spiritual knowledge and traditions into account.

**Supplementary Information:**

The online version contains supplementary material available at 10.1186/s12904-024-01431-5.

## Introduction

Indigenous peoples often face barriers to accessing culturally safe end-of-life care [1]. These constraints may be compounded in rural and remote settings where health services in general are limited [2, 3]. Indigenous peoples’ values, cultural knowledge, rituals, and expectations of care related to death and dying may differ from mainstream healthcare providers and health services [5, 6]. This tension can lead to different expectations, goals of care [3, 7] and differing understandings of what death means (i.e., a definitive end-point vs. a spiritual transition) [2, 5, 8]. Where there is a lack of understanding, trust or communication between the community and health service institutions, Indigenous peoples may not feel safe to receive mainstream palliative care services [3, 9, 10].

There are variable perspectives on the meaning of “palliative care” and Indigenous peoples often prefer alternative language, such as “care of the terminally ill” and “preparing for the journey” [6, 11]. This article refers to end-of-life care as comfort care provided to a person who is in their last days of life within the wider category of palliative care, which can be provided to a person with a life-limiting illness, even if they may have a year or more left to live [6, 11]. There are limitations in the scope of each term, and although “end-of-life” offers greater specificity when desired, we recognize that it also implies a finality to death which the Innu worldview does not support. Nevertheless, “palliative care” remains a common term in healthcare settings, and so we have retained its use when specifically referring to healthcare services that identify as palliative.

Research has shown that Indigenous peoples often wish to die at home, rather than in hospital, where family and community members can provide support and care, and help ensure shared decision-making in their approach to end-of-life care [1, 3, 12, 13]. However, non-Indigenous, mainstream healthcare systems often depend upon hospitals to provide end-of-life care services in rural and remote Indigenous communities [2]. The physical structure of hospitals and visiting policies may limit access to family supports [3, 12], which can create conflict between communities and health care providers, and hamper the quality of care Indigenous peoples receive. This inequity is further complicated by the history of racism towards Indigenous populations in Canada. For example, survivors of the residential school system may experience a hospital admission as a return to previously traumatizing institutionalization [9]. Non-Indigenous persons often prefer to die at home, and the balance of dependence on community and culture pitted against mistrust of the hospital system is starker in the face of Indigenous people’s historical experience of systemic racism.

In this and other ways, Canadian health systems often fail to meet the cultural and spiritual goals of care of Indigenous peoples in end-of-life care. Indigenous peoples often have a holistic understanding of health and wellbeing, which involves wellness and strength not only in the body, but also in the mind, spirit, and relationships [4, 5]. Traditional healing practices and the participation of community members, particularly Healers and Elders, should be integrated into the process [4, 8]. Therefore, culturally safer end-of-life care for Indigenous persons requires communication by physicians, families, and interpreters [4, 14], done in a respectful way [11], with adequate time given to establish trust [4, 12, 14, 15]. The process requires sufficient space and resources to address language and other cultural barriers that increase the risk of misunderstanding and isolation [4, 12, 16–18].

As described in the Improving End-of-life Care in First Nations Communities (EOLFN) workbook, the first phase in developing palliative care programs in First Nations communities in Canada is “grounding the program in community values and principles” [19]. Therefore, to create culturally safer end-of-life care services, healthcare leaders must listen carefully to and collaborate with the Indigenous communities for whom they aim to provide service [8]. To this end, in 2016, Innu community leaders from Sheshatshiu, Labrador identified a need for research to inform health service planning related to end-of-life care for the Innu. The project is the result of a collaborative community partnership between the Innu Nation and Memorial University’s Faculty of Medicine, with support from the Sheshatshiu Innu First Nation (SIFN) Band Council, Memorial University’s Labrador Campus, Labrador-Grenfell Regional Health Authority, and the Department of Health and Community Services, Government of Newfoundland and Labrador.

Throughout this project, we have endeavoured to adopt the lens of cultural humility, a quality of healthcare service in which the healthcare provider (individual or institution) reflects upon their own culture and power relative to their patient interactions and patients’ context, with the goal of providing culturally safer care [12, 22]. Cultural humility, therefore, is a pre-requisite to providing culturally safer care. This is different from language of cultural competency, which implies mastery of certain information about a culture [20, 23]. Culturally competent strategies risk essentializing minority groups and acting upon stereotypes [23, 24]; they may or may not contribute to cultural safety, depending on how and when they are used [12]. Therefore, many have called for a progression from cultural competency to cultural humility and safety [12, 20, 23, 25]. Culturally “safe” care is an outcome experienced by the recipient of the care and is not a quality which the service providers themselves can judge. For this reason, we refer to culturally “safer” care when addressing the provider’s delivery of care rather than the recipient’s own experience of the care [26].

The purpose of this study was to advance knowledge about end-of-life care to inform culturally safer end-of-life care practices. Specifically, we aimed to:

1. Describe the cultural and spiritual knowledge and practices related to death and dying of the Innu in Sheshatshiu, Labrador;
2. Identify aspects of current end-of-life care delivery that serve the cultural and spiritual needs of the Labrador Innu as well as those that fail to meet these needs; and
3. Explore ways to integrate current end-of-life care delivery practices with Innu cultural and spiritual practices to achieve more culturally safe care delivery.

## Methods

### Design

We conducted an exploratory, qualitative study using semi-structured interviews and a focus group to understand the spiritual and cultural end-of-life care practices of the Innu in Sheshatshiu, Labrador. Our methods have used an inductive thematic analysis approach [27] and incorporated tenants of Community-Based Participatory Research through close collaboration with the Indigenous community [30]. We used the Consolidated Criteria for Reporting Qualitative Research (COREQ) checklist to report the findings [31].

### Participants

Interview and focus group participants were recruited using purposive sampling to ensure we included those with experience related to end-of-life care. All participants were identified by an Innu Advisory Committee (IAC). Eleven interviews were conducted with healthcare professionals who had a role in providing end-of-life care in Sheshatshiu (nurses, *n* = 2; family physicians, *n* = 3) and leaders with a role in making policy and program decisions related to health services in the community (decision-makers, *n* = 6). Decision-maker participants included representatives from the Innu Nation, the Sheshatshiu Innu First Nation Band Council (SIFN), the Innu Round Table Secretariat, Labrador-Grenfell Regional Health Authority, and the Government of Newfoundland and Labrador. One focus group was conducted with Innu Elders (*n* = 5) from Sheshatshiu. The total sample size was 16 participants (Table 1), of which 50% were Innu and 62% were women.

**Table 1:** Study Participant Demographics

Role	Number of Participants	Innu	Sex
Elders	5	5 Yes, 0 No	4 Female, 1 Male
Decision-Makers	6	3 Yes, 3 No	3 Female, 3 Male
Healthcare Professionals	5	0 Yes, 5 No	3 Female, 2 Male
Total	16	8 Yes, 8 No	10 Female, 6 Male

### Data collection

Once identified by the IAC, the interview and focus group participants were invited to participate by members of the research team. Innu Elders and Innu decision-makers were invited to participate by JP. Healthcare professionals and non-Innu decision-makers were invited to participate by RD, MvS or HH, depending on who had the most appropriate working relationship with the person. Participants were invited by either email, telephone, or face-to-face invitation, depending on which method the team felt was most culturally appropriate for the participant. Oral or written consent was obtained for all participants.

Interviews were conducted by three of the authors. RD is a male family physician with a graduate degree and is Associate Professor at Memorial University, having completed approximately 30 qualitative interviews in the past. MvS and HH are both female family physicians undertaking or having recently completed a postgraduate enhanced skills program in Care of Underserved Populations, which included previously working in the community with the participants they interviewed. The interviewers were not Innu themselves and so they reflected upon their own positions, perspectives, and potential biases and how they might shape the research. To ensure Innu interests, perspectives, and experience were captured meaningfully, the interviewers collaborated with the IAC to determine the interview process – question guides (developed for this study), setting, and language where appropriate – as well as throughout the data analysis and interpretation processes. Separate question guides were created for Innu community members (i.e., Elders in the focus group; see Supplementary File 1) vs. decision-makers (see Supplementary File 2) vs. healthcare professionals (see Supplementary File 3), each with 4–8 open-ended questions (plus follow-up probes, as needed) regarding Innu culture, experiences with the current healthcare system, and Innu needs concerning end-of-life care.

MvS and HH pilot tested the interview questions with a non-Innu community member, and RD reviewed the recording to provide feedback to the interviewers on their technique as well as the interview question guides and prompts. Interviews were conducted between May-September 2020 at a place of convenience and safety for each participant, either by telephone or in-person, typically at a private location in the workplace. The duration of the interviews was between 25 and 80 min. No interviews were repeated, but specific points of clarification were sought in follow-up by email or in conversation.

RD and JP facilitated the focus group held in September 2020 in Sheshatshiu, Labrador. The session lasted approximately 2 h and 20 min. An experienced translator from the community was available to provide simultaneous interpretation so that participants could express themselves in their chosen language. A family medicine resident from Memorial’s Care of Underserved Populations Enhanced Skills Program also attended the focus group as a note taker.

Both the interviews and focus group were audio-recorded and transcribed verbatim. Field notes were also taken during the focus group to capture relevant contextual information and other observations, such as group dynamics, that might influence analysis. All interviews were conducted and transcribed in English. The focus group transcript captured the real-time English translation of discussions, but the Innu-aimun spoken during the focus group was not transcribed. Interviewing and analysis was iterative. The research team met to discuss ongoing interviews and emerging themes and decided that data saturation had been reached after these 11 interviews and one focus group were completed.

### Data analysis

The interview and focus group transcripts with field notes were analysed using Braun & Clarke’s inductive thematic analysis approach, as outlined step-by-step by Maguire & Delahunt [27, 32]. First, MW and RD independently coded the same three transcripts using Microsoft Word and then met to achieve consensus by comparing initial codes and discussing the coding of difficult passages. Through multiple meetings, RD and MW developed a thematic tree using pen and paper to group the codes and then produced an initial codebook in Microsoft Excel. MW recoded the original three transcripts as well as three additional transcripts with the initial codebook and met with RD to discuss any new and/or revised codes, review any passages that were difficult to code, and update the codebook accordingly. RD travelled to Sheshatshiu and reviewed the codebook with the IAC. The IAC made comments and revisions that were incorporated into the codebook, including, for example, suggesting a code concerning the cultural significance of the Innu-aimun language. MW coded all transcripts using this codebook, discussing any difficult sections and new and/or revised codes with RD. This iterative process produced 5 further revised versions of the codebook over the course of one month, with mostly minor revisions each time. The final version of the codebook included 4 levels of code: 3 first-level codes (i.e., cultural needs and strengths; services; and organizations’ relative roles), 15 second-level codes (e.g., visitation; location; strengths of Innu culture; etc.), 62 third-level codes (e.g., who needs to be there; what needs to happen; family-centered; etc.), and 104 fourth-level codes (e.g., community, Elders, sharing stories; etc.).

As coding progressed, MW began to explore emerging themes in collaboration with RD. RD sought input from the IAC members on both the codebook and preliminary themes during his visits to Sheshatshiu. During one such visit, emerging themes were identified around (1) what a good death looks like for the Innu, (2) characteristics of an effective end-of-life service for the Innu, and (3) which people need to be involved in the end-of-life process and how they work together. These were discussed and received further feedback from the IAC. When analysis was nearing completion and main themes had been identified, RD, JP & HH presented the themes at a Town Hall in Sheshatshiu with IAC members in attendance, to invite community dialogue and feedback on the themes. A virtual attendance option was provided so that participants outside Labrador could also join the discussion. Community members engaged with the findings and contributed to a rich discussion about their relevant experiences. The research team considered the community’s discussion and feedback and determined that no major changes to the codebook or themes were warranted. Participating Elders asked that quotes be attributed to them by name, in recognition of their expert contributions.

## Results

**Fig. 1:**
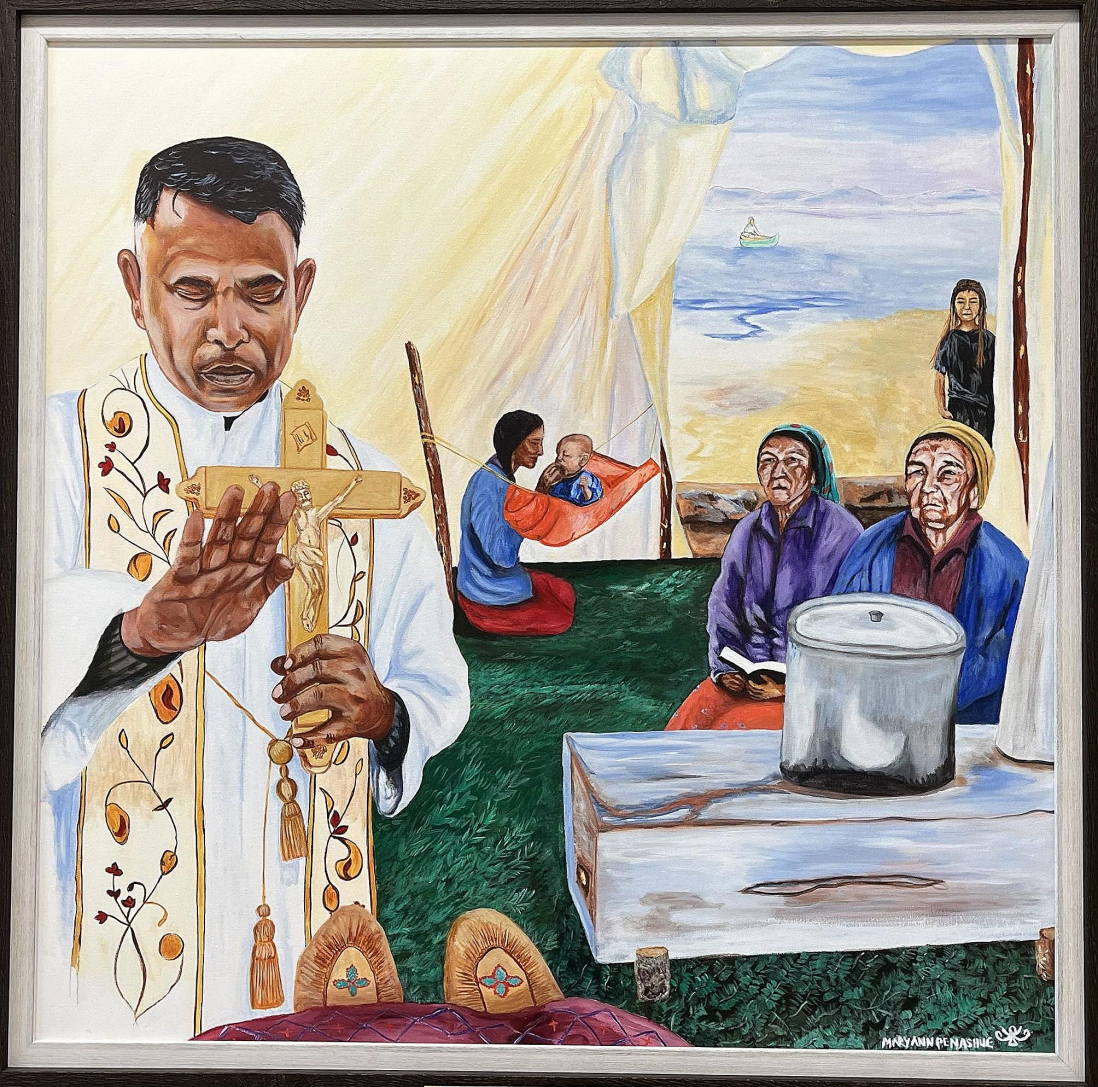
Painting representing our findings of Innu Elder approaching end-of-life in a tent (by Innu artist Mary Ann Penashue)

We identified seven themes highlighted below capturing issues related to Innu cultural practices pertaining to death and dying, current approaches to end-of-life care, and ways to support culturally safer practices in end-of-life care. Our findings are also represented visually in a painting commissioned from an Innu artist in Sheshatshiu (see Fig. 1), as this was felt to be a more culturally appropriate form of knowledge translation for the Innu than written text. Two original copies of this painting were created, one for Innu Nation to display in Sheshatshiu and one for Memorial University to display in the medical school in St. John’s.

### Relationships and visitation support a “peaceful death”

The Innu of Sheshatshiu view dying as a collective process that happens best within the community. The strong relationships among immediate and extended family, and with the broader community members, are strengths of the Innu culture, and are foundational to the end-of-life experience for Innu.

A key feature of those who “died peacefully” (Rose Montague, Innu Elder) includes visitation from family members and friends, Elders, community leaders, and others in the community such as priests and healthcare providers. Elizabeth Penashue, an Innu Elder, describes the death of another Innu Elder:

She was probably happy to be home with her family surrounded by friends and family. So, she was able […] to hear talking, telling stories and serving tea and it was a peaceful way for her last request to be at home with family and friends you know gathered around. So that’s a peaceful way to go.

An Innu decision-maker in the community describes a “peaceful death” for Innu in a similar way:

He or she was very sick and there’s a lot of people gathered even those people they’re not related they’re still going same place trying to comfort this family and the children of the Elders and I think it’s part of our culture because they’re always try to help one another. These people that’s what they are, that’s what they do and that’s what they like to do….

Rose Montague, an Innu Elder, talked about spending time with a loved one as they were dying:

[W]hen she knew that [her mother] was really sick and won’t have much time to live her mother would say ‘don’t leave me.’ You take turns, like the family, take turns being there for her and the same thing with her husband you know we don’t want to leave, we didn’t want to leave him. We wanted to be there for support.

Participants emphasized the importance of Elders being present for Innu who are dying. Elders are able to provide comfort, share stories, and lead cultural and religious ceremonies such as communicating with families, providing spiritual support, and preparing traditional medicines. Theresa Andrew, an Innu Elder, commented:

It’s important to have an Elder there because […] an Elder knows what’s important. Like an Elder will automatically think of a priest, to call the priest to come and say their last you know the prayer. It’s just, an Elder is very religious so that’s why it’s important to have an Elder there.

Joseph Mark, an Innu Elder, described the role of traditional Innu medicines for those approaching the end-of-life,

There are medicines out there, there are medicines out there you know medicines that you can use when a person is sick but when they don’t have much time to live then there’s not much you can do.

Healthcare providers are also seen as a welcome source of support, beyond providing medical information and treatment. Providing comfort to the both the individual and their family is key. Elizabeth Penashue, Innu Elder explained,

It would be helpful if…the doctors or the nurses were also there present when the person is you know on their death bed or passing rather than just bring the news to the family and then leave. It seems like they bring the news and they can’t do very much now so then that’s it they leave the room. It would be very good if they were there to be supportive and be there when a person is passing.

### Traditional locations of death and dying

Location of death and a connection to the land are also very important, as emphasized by many focus group and interview participants. For the Innu, dying in the community, either at home, in a traditional Innu tent, or “out in the country” (as Elder Elizabeth Penashue used the phrase in reference to the Innu traditional way of life on the land) is much preferred to dying in the hospital. Where Innu die helps to sustain a connection to the land, which is a core aspect of Innu identity, culture, and spirituality.

Francesca Snow, an Innu Elder, talks about this:

Hospitals is not the best way to die. I think it’s best for you to be very peaceful […] Like my dad he was about 98 or 100 years old. He wanted to die at home. So they took him home and he died there peaceful at home. And he was alive for at least a week before he pass away and that was a good feeling for all my family visiting and cooking and all that […] I really think all people wants to die at home.

Elizabeth Penashue, Innu Elder, described her husband passing away in a tent,

When my husband passed away he died in a tent so we had requested that he pass in a tent and he said it was a peaceful passing and he was comfortable, he was surrounded by family and I am sure that is what he wanted to see and you know passing in – taking his last breath in a tent surrounded by family.

Permission to include this figure granted from the artist, Mary Ann Penashue [33].

### The important role of friends and community in providing care

End-of-life care for the Innu in Labrador is provided by family, friends, and other community members, a local interdisciplinary healthcare team, and by hospital services in Goose Bay.

Family members are usually the primary caregivers. They are responsible for meeting the dying person’s everyday needs and often arrange or advocate for their end-of-life medical care. Family members are supported in these tasks by the community at large. Clergy and Elders provide spiritual support to the dying person and their family. An Innu decision-maker talked about the many people involved in caring for community members who are dying:

If people are going through tragedy or death or somebody is sick there’s like there’s always people at their house supporting, cooking, talking, and praying and there’s probably 20 or 30 people there being supportive to the people that are going through it.

Though the family may take a central role in the delivery of end-of-life care at home, they often work very closely with the local interdisciplinary team of nurses, family physicians, and home care workers. When a patient is identified by the interdisciplinary team as needing an end-of-life care discussion, a family physician will complete an assessment and referral to the Population Health Program for further end-of-life care, if appropriate. At times, end-of-life care services and supports in the community may be arranged informally, relying upon relationships that exist between families, healthcare providers, and community supports. For example, some of the nurses based in the community clinic have a long history of working in the community which builds trust and, in turn, improves delivery of care. However, an informal arrangement has limitations, and is largely dependent on the goodwill of individuals to deliver those services.

[T]he physician would generally do a home visit and often times that would be done with the RN from Goose Bay from the End-of-Life Program and then […] the doctor would write all of the [medical] orders. The RN would take the orders to Goose Bay, get the medications, come down, do a whole bunch of teaching with the family on how to administer the medications, put in some subcutaneous [injection ports for] whatever the meds were to be given and leave them with the family and then kind of check in on a daily basis or twice a day or whatever they kind of agreed on. (non-Innu Healthcare provider)

In some cases, end-of-life care is delivered at the Labrador Health Centre in Goose Bay, approximately 40 km from Sheshatshiu. End-of-life patients at the Labrador Health Centre receive care from an interdisciplinary team consisting of physicians, nurses, social workers, and physiotherapists, for example. Patients also have access to spiritual supports including a chapel and clergy. An interpreter is also available to assist with translation services.

### Flexibility and communication regarding cultural practices

Local healthcare providers and decision-makers believe that adopting a flexible attitude to find innovative ways to prioritize cultural needs and open communication about end-of-life care demonstrates respect for Innu persons and a desire to provide culturally safer care.

Participants talked about some important examples of how healthcare providers have successfully worked with Innu families and community leaders to integrate Innu cultural practices into end-of-life care. In addition, the Regional Health Authority is designing a larger, dedicated Indigenous space within the hospital, in partnership with local Innu and Inuit communities, that they believe will be helpful during the end-of-life care process.

I can remember at one time this lady was in palliative, she had died, and the ladies arrived, and it was in the old hospital not the new one. So, the nurse in charge provided them with a room where they came with their sewing machines […] and they went in the two or three ladies and made moccasins and shrouds and everything in preparation [for the funeral]. (non-Innu Healthcare provider)

I especially remember my dad he was in Intensive Care Unit and there’s a lot of people and there’s other patients lying there and there’s doctors and nurses and so on and there’s other people. Really big traffic. It’s just like a cafeteria [-] there’s really a big lot of people […] but they manage to move my dad; I think that’s when they know that […] he wouldn’t be able to make it so they move him to another room which is a bigger room, but is [usually used as] a nursery room for the babies. So that’s where he passed because it’s a bigger room…. [T]hank God that like doctors and [the health authority] they try to do the best that they can but they don’t want to ask the family to go as well because of this really difficult time. It’s not just my father’s situation, I see [it] all the time. (Innu Decision-maker)

Healthcare providers, Elders and Decision-Maker participants all commented that, when open communication around end-of-life care takes place in the early stages of a terminal illness, it improves the quality of care received by patients and their families. They reported that when they can discuss goals of care early in a terminal illness, it provides the opportunity for the provider, patient, and family to discuss and plan for cultural needs and expectations surrounding end of life, which can help health providers and the health system to understand their needs and take steps to meet them. A non-Innu decision-maker from the region described an innovative example:

It was the wish of that particular Elder to pass away in a tent and that person would have had the experience of living on the land and previous to actually living in Sheshatshiu and so … the tent was set up here at the health centre so that the providers could have that ease of access. So … the staff there were able to, whether it be medications or assessments or just in comfort measures, were able to actually go and provide that to that individual right there on site while also maintaining what was important that individual with regards to being in the tent.

Innu Elder, Francesca Snow, talked about the need to discuss patient wishes about interventions at the end of life:

I think it’s very important that people know you want to revive him or you don’t want to revive him and not be in there for request of paper being signed by the person or the family member that might – when my dad died he don’t want to be revived. So as a family member we have to respect that, and maybe other members want to revive so there’s a difference and that’s why doctors should be present at that time.

### Adequate and appropriate supports and services

Current end-of-life care sometimes fails to adequately support patients and their families during and following a death. The Innu are often not able to access the services they require in their home or community, communicate effectively with healthcare providers in their own language, and at times they experience poor continuity of care. Participants attributed some of the challenges with continuity to having a transient health workforce with many providers that have limited knowledge or experience with Innu culture. Some participants stated that, despite the importance of dying at home with family, this is often a difficult scenario to support. Sometimes, this is due to lack of adequate space and resources in the community. Other times, it may be a result of late discussions regarding discharge planning from hospital.

…she thinks about her brother who had passed away and there was so many of them in you know trying to visit and so it gets overcrowded with you know Innu always trying to be there for one another. (Rose Montague, Innu Elder)

…they died alone in the hospital and they wanted to get out, they were getting ready to because they said they didn’t want to die alone and they wanted to die at the house if the family members can’t be there with them and before that could be arranged to get them home they just died and so that’s sad because Innu people […] have to have people, they like having people around and so it’s been a challenge… (Innu Decision-maker).

Francesca Snow, Innu Elder, highlighted the potential increased risk of suicide, in the absence of supportive services, when community members are struggling with grief over the loss of a family member:

Some of us probably would be suicidal and that’s very important. I have families who committed suicide […]. A lot of people lack with counselling. They never even have it and that’s why it’s very important for the family to have and that would satisfy me. When they are in the hospital, they have nothing.

Communication in the Innu-aimun language facilitates the delivery of end-of-life care through culturally safer communication around sensitive topics and helps the Innu maintain a sense of connection to their cultural identity. However, most healthcare providers serving the Innu of Sheshatshiu do not speak Innu-aimun and must depend on an interpreter. Participants noted that the hospital’s one interpreter works in multiple departments and is not always available. To improvise, patients often rely on family or friends, usually younger people, to translate for them. This situation may lead to privacy issues as well as communication barriers, which can be particularly difficult to navigate when it comes to sensitive topics and informed consent around medical interventions.

I think it’s so hard for the Elders, much harder for them than us […] the Elders only know their [own] way [….] [W]hen they’re in the hospital they don’t speak the language, they don’t understand the language. Like I speak it and I understand it but there’s another language to the medical terminology and I don’t understand that I have to say, ‘What’s that or what’s this? Can you say it the other way so I can understand what I’m doing here?’ But [the Elders] can’t even say that for themselves and if somebody is not there with them that’s probably harder when they have to deal with that. (Innu Decision-maker)

I think we’re really lacking on the interpreter resource. I think we often do without it and that’s probably not good enough often using family or others and because we don’t have a consistent interpreter resource here […]. You know often with the Innu when they’re dying in hospital there are a lot of people around so there are people to communicate with but it’s not always clear who is the best, who should be communicated with about the patient or if you can’t communicate directly with the patient. Yeah so, we don’t have a whole lot of resources in that way and we really depend on families which I don’t think is ideal. (non-Innu Decision-maker)

Participants reported that the healthcare workforce in Sheshatshiu is often understaffed and transient. There are not enough care providers such as physicians, nurses, home support workers, or interpreters to serve the region adequately, and those present often lack familiarity with the Innu culture. The lack of consistent access to care and support in the community is difficult for patients. Adding new providers during end of life does not provide Innu persons and family members adequate time to get to know them and establish trust.

A lot of times people are agreeable to take care of a loved one during this palliative care time and then all of a sudden it gets too frightening and they know you can call an ambulance and they will be taken to the hospital and looked at and it does happen a few times. (non-Innu Healthcare provider)

[T]here tends to be lots of turnover and they may not be tuned into the different needs of our Indigenous People. (non-Innu Healthcare provider)

### Culturally-informed policies and leadership

Participants emphasized the need for the health system to develop culturally-informed policies and guidelines that support providers and health facilities to meet the needs of Innu persons and communities.

Innu participants express the need for agency and control in the essential services provided to their people. Innu participants explained that when Innu persons do not feel a sense of agency and their cultural needs are not integrated into the end-of-life care, families and the community view their contact with the health system as a continuation of the medical colonization that has shaped their historical and contemporary experiences with healthcare.

I think wherever Innu are going to get the services they should get advice from Innu people and what they do in their own ways or Innu way and try to implement that in their policies or in the system or to get like we’re not stupid, we know what we need and what we need to do it’s just that sometimes it’s not, I don’t know what it is but I think it’s all about power and control yeah and like that’s that used to be back in a day when kids were taken away they would be at the hospital and all of a sudden they’re not there because they’re sent away and get adopted out and there’s nothing you can do; it’s the government. (Innu Decision-maker)

Innu participants stressed the importance of having Innu representation on the Regional Health Authority’s governing board. However, the process for recruitment to this board is not always well understood. The board is overseen by an Independent Appointments Commission with a provincial mandate, that the Health Authority itself does not control. Healthcare decision-makers echoed the value of having Innu representation on the health board.

We would really like to have an Innu Board member or an Innu Board members I think that’s really important for the for our health authority. […] We have nothing to do with that, so the province has an […] Independent Appointments Commission which vets Boards and Board Chairs for all Boards in the province. So health boards, school boards, all boards are basically appointed by that Independent Appointments Commission and it’s a bit of a process. […] We don’t know who applies or what happens in there. We get assigned a board right. So we get a letter from the Independent Appointments Commission saying these are your new Board members right and so we have no role in that at all, but you know there absolutely should be Innu representation at our Board. (non-Innu Decision-Maker)

Hospital policies and regulations can limit the ability of the Innu to experience end-of-life in ways that meet their cultural and traditional needs. While policies such as these often have good logistical reasons behind their initial creation, they can be culturally harmful to the Innu if uniformly applied in all situations, regardless of the circumstance. For example, gathering of family and friends and visitation with their dying loved one is a very important element of end-of-life care for the Innu but it is not always accommodated in hospital settings.

They had wanted to gather all of the extended family around and sing hymns and say prayers. And the family was so large that they were kind […] spilling out into the hallway while they were singing, and they were actually all asked to leave and go the chapel. And I think the people that asked them to leave like I’m sure they didn’t mean to come across you know disrespectful, but the family took it very disrespectful and they were very, very upset with their experience that they had and that patient ended up dying and they all weren’t present. (non-Innu Healthcare provider)

Decisions about where services should be provided need to involve the Innu. For example, Innu participants identified that Sheshatshiu needs a hospice where end-of-life patients and their families could access services within the community. Offering this service requires planning for adequate space and resources. Supports like a grief circle after a loved one has passed away can provide important aftercare to family members and give them someone to talk to about their experiences and feelings.

…it would be really good to have our own building for such things like this because of you know she thinks about her brother who had passed away and there was so many of them in you know trying to visit and so it gets overcrowded with you know Innu always trying to be there for one another. (Rose Montague, Innu Elder)

All you need is a building and good resources in it, good resources where a lot of people could come in and pray like I said our tradition is prayers and be with the family…and also counselling the people. (Francesca Snow, Innu Elder)

Healthcare decision-makers reported the need to collect feedback about healthcare services in a culturally appropriate way. In particular, tools such as survey questionnaires or client feedback forms may not be accessible methods for many Innu. By contrast, well-established relationships and open lines of communication with Innu community leaders can create informal opportunities for meaningful feedback. It is important for healthcare decision-makers to “communicate in ways that make sense to Innu First Nation.” (Decision-maker, non-Innu).

[W]e know that some of the formats that we may take for granted in some populations you know like filling in surveys and that sort of thing that doesn’t seem to be culturally [effective]. I find that listening [to] things that come through the [Innu] Chief or you know work that might come through the navigator […] for example and that sort of thing, some of that works. (non-Innu Decision-maker)

Our findings show that, although cultural training is very important, most healthcare providers do not receive any. Participants feel that immersive experience is a vital part of learning cultural safety: living in and working with the community, learning from Elders, going out to the country with the Innu, etc. This kind of immersive training works best when care workers are based in the community for a longer period of time and are committed to understanding the culturally-specific care needs of the populations they serve. Immersive learning would ideally complement cultural safety training for healthcare providers.

Similarly, improving communication between healthcare providers and Innu persons and their families is also an important aspect of providing culturally safer care. The Innu culture is closely related to the Innu-aimun language and many Elders do not speak any other language. Therefore, ensuring timely access to appropriate interpretation services, both in the community and in the hospital, is foundational to protecting the cultural safety of these patients.

Interpreters is the biggest thing I feel. Well maybe not the biggest thing but it’s certainly one of the first things that comes to mind because we do find that there is often a lot of confusion between what the family has heard from the staff at Lab Grenfell to when they get home what they’re expecting to happen and what that actually looks like there’s usually a lot of confusion and miscommunication and I do believe that it’s simply because it’s not translated appropriately simply because the doctor or whomever will go I and say well do you understand and the family just says yes or the client says yes and really they’ll come back and they’ll call and they’ll be like the doctor gave me all these papers and I don’t know a thing and then we will go through them. (non-Innu Healthcare provider)

### Innu care providers and patient navigators

The health system should include Innu care providers and/or navigators in the circle of care.

Healthcare decision-makers and healthcare providers identified a need for Innu care providers who are best equipped to communicate with patients and families, both in the Innu-aimun language, and through their own experience with the culture.

It would be great if we had local Innu nurses who could come in and speak the old language and discuss all of the end-of-life things with them [Innu patient and family] in their language because sometimes there is things that are lost in translation to especially when you’re having those important conversations so that would be an asset for sure. (non-Innu Healthcare provider)

Providers and decision-makers have also suggested an Innu patient navigator at the hospital is helpful to support culturally safe experiences of care for Innu persons. The navigator would also communicate patient concerns through appropriate channels and act as a spokesperson for the family in hospital and community settings.

I think ideally there would be somebody like 24/7 at the hospital […] who is simply there for that like a patient navigator type of person like you would have in St. John’s. I think we need the same thing here. (non-Innu Healthcare provider)

…the Innu navigator position [is intended to] support people when they need to navigate the system and when they have concerns about their care. We have established that piece of the navigator being able to act as a spokesperson with the permission of the individual if they have concerns about their care. (non-Innu Decision-maker)

### Summary

This qualitative study explored the end-of-life care experiences and perspectives of Innu Elders, healthcare providers and decision-makers in Sheshatshiu, Labrador to better understand the end-of-life care needs of the Innu of Sheshatshiu, and to highlight how well the current medical and community services meet those needs. We found that a peaceful death for the Innu requires the presence of family, Elders, and many others from the Innu community, as well as a strong connection to the land, preferably taking place at home instead of in a hospital. Interviews revealed that while the current end-of-life care service provides an interdisciplinary healthcare team for the community and hospital services in Goose Bay, there is heavy reliance on support from family (the main caregivers) and community members. Because of this, the need for translation services is critical. When healthcare providers and decision-makers maintain a flexible attitude and open, timely communication, this helps facilitate culturally safer care. Unfortunately, lack of services (e.g., translation, counselling) and a transient workforce of healthcare providers contributes to inappropriate outcomes, such as Innu persons dying alone in a hospital against their wishes. This study suggests that to achieve culturally safer care, the Innu need to be directly involved in policy development and leadership roles within the healthcare system. Additional resources were called for, including meaningful cultural training for healthcare providers, formal interpretation services, a community hospice in Sheshatshiu, an Innu patient navigator in the hospital, and the recruitment of care providers who are themselves Innu.

## Discussion

Our findings are largely supported by existing literature on end-of-life care for Indigenous peoples. Similar to our findings concerning the role of family and community in end-of-life care among the Innu of Labrador, existing literature [3, 12] also emphasizes the important role of informal care provided by family, friends, and other community members in end-of-life care among Indigenous persons. However, Habjan et al. suggested that increasing outmigration of young people from Indigenous communities may lessen the support and respect they provide to community Elders, leading to an increased reliance on formal healthcare programs [1].

Studies have also echoed our findings of the importance of services provided within the community, thereby maintaining a connection to the land [6] while providing adequate local infrastructure [11, 13, 34], such as a hospice or friendship centre, in which members of the community can gather and provide support for community members nearing the end of their life [3, 20]. These are essential to building local capacity and “community readiness” to provide end-of-life care [12, 19, 35].

Several authors also stated the importance of an Indigenous patient navigator or “case manager” [20, 36], with Fernandez et al. (2010) finding this to be the most-used support role in their multidisciplinary home-based palliative care program [36]. Nadin et al. (2018) piloted an end-of-life care program which included an on-call care coordinator and found anecdotal evidence suggesting that the coordinator helped to prevent unnecessary use of health care services [13]. By contrast, Fernandez et al. (2010) did not find a decrease in emergency department visits, but did find a statistically significant reduction in acute care hospital admissions. Altogether, this may suggest home-based palliative care programs with a case manager are not only culturally safer, per our findings, but also cost-effective [36].

Despite many similarities, there were differences as well. For example, we (like Kelly et al., 2009 [34]) found people valued a direct discussion about expectations and goals of care, whereas Nadin et al. (2018) found that within their community “talking about death and dying is not culturally appropriate” (p4-5) [13]. Such diversity further emphasizes the importance of any end-of-life care program being designed to fit the local Indigenous community’s context [6].

### Implications for clinical care and policy

This study shows that the history of racism and the resulting intergenerational trauma have had a lasting impact on how the Innu experience and perceive the care they receive when those in power have not engaged them in the development of those services and policies. The degree of trust a community inherently feels towards healthcare providers and other authority figures may vary among individuals, groups, and cultures. Where trust has been damaged, rebuilding is required on the part of current providers and other health system leaders. This can be done through consultation with the Innu, including leadership from the Innu community in planning their own care and services. Indigenous peoples often feel frustrated and disempowered when governing bodies do not appear to take their input seriously [1]. Therefore, those in power must reflect on the ways that policy development and health system leadership, currently often based on a mainstream business/governance model, present cultural barriers to effective Innu participation.

Culturally safer policies should also integrate traditional healing practices and beliefs. However, the study findings show that some healthcare providers are more open and willing to incorporate these practices than others. Culturally safer policies and services should therefore consider how these traditional practices can be integrated into patient care. Furthermore, institutions should support their employees to integrate the inclusion of traditional practices into the care they provide.

Culturally safer policies can only be implemented if the resources are in place to support them. For example, there is a need for consistent access to Innu translators and care providers who are skilled in bridging the gap between Innu cultural knowledge and mainstream medicine. Such systemic changes need to go hand in hand with collaborative efforts between the various institutions and stakeholders responsible for the care of this community, so that the necessary resources (human, educational, structural, and financial) are provided and maintained. Jurisdictions between organizations involved in providing end-of-life care to Indigenous communities are often unclear, in which case increased communication efforts and/or guiding policies are needed to ensure the communities’ needs do not slip through the cracks [6].

### Implications for future research

If our findings are incorporated in any future changes to the end-of-life care services available to the Innu of Sheshatshiu, then it would be beneficial to evaluate those changes/services (e.g., Nadin et al., 2018 [13]) to determine their impact and describe any enablers and barriers to success that are encountered. Additionally, a better understanding is needed of the various stakeholders/organizations’ relative roles and mandates, as well as how Innu and non-Innu leadership can most effectively collaborate in palliative (and general health) care provision. Given this study’s focused setting, further study in other Innu communities (e.g., Natuashish in Labrador and communities in Quebec), and other Indigenous populations in their unique contexts is warranted to see where the above themes translate and where they differ. Investigations will be needed to inform healthcare professionals and services who seek to provide culturally safer care to other diverse Indigenous populations. Ultimately, it is for the respective Indigenous communities themselves to identify what questions they feel should be studied in their context and what future research is appropriate for them.

### Strengths and limitations

The primary strength of this study is the relevance of the topic to the community’s own identified needs and the high level of engagement from the IAC throughout the project. The specific geographic location in additional to cultural differences among Indigenous peoples limits the transferability of these findings to other settings. However, transferability of findings was not our primary aim. The study was conducted in a focused context, so that the findings are directly relevant to this population and the palliative care services provided to them. Nevertheless, the alignment of our findings with previous work showing similar themes related to the nature, location, and family involvement in end-of-life care speaks to the commonality and importance of these issues to Indigenous end-of-life care. The findings are further strengthened by the immersion of the nominated primary investigator (RD) in all stages of the study ensuring consistency and congruency through data collection, analysis and writing. Finally, the study findings were generated with meaningful and continuous engagement from the Innu community in Sheshatshiu, as per Article 9.17 of Chap. 9 (Research Involving the First Nations, Inuit, and Métis Peoples of Canada) in the Tri-Council Policy Statement: Ethical Conduct for Research Involving Humans (TCPS2) [37]. In this way, the community has worked with researchers to ensure the accuracy of the data they provided (respondent validation). This was critical in ensuring that the voices of the Sheshatshiu Innu community were adequately and respectfully represented.

## Conclusions

The Innu in Sheshatshiu have a rich culture that contributes to the health, care, and overall well-being of Innu people approaching end of life. Key results from this study include the Innu’s strong ties to family, friends, community, home, and the land. Mainstream medicine is often beneficial in the care that it provides; however, when this care is provided without regard for the traditional needs of the Innu, then it can do harm. The results of this study point to ways in which the healthcare system can work with the community to provide culturally safer end-of-life care to the Innu of Sheshatshiu.

### Electronic supplementary material

Below is the link to the electronic supplementary material.

Supplementary Material 1

Supplementary Material 2

Supplementary Material 3

## Data Availability

The datasets generated and/or analyzed during the current study are not publicly available as they are the property of Innu Nation on behalf of the Innu community of Sheshatshiu. However, data are available from the authors upon reasonable request and with the permission of Innu Nation.

## Declarations

### Authors’ information

This research collaboration is the result of a community partnership between the Innu Nation and Memorial University’s Faculty of Medicine, with support from the Sheshatshiu Innu First Nation (SIFN) Band Council, Memorial University’s Labrador Institute, Labrador-Grenfell Regional Health Authority, and the Department of Health and Community Services, Government of Newfoundland and Labrador. An Innu advisory committee was formed at the beginning of the project to guide all stages of the work.

## Abbreviations

SIFN: Sheshatshiu Innu First Nation
IAC: Innu Advisory Committee
