# Supplementary material for: Patshitinikutau Natukunisha Tshishennuat Uitshuau (a place for Elders to spend their last days in life): a qualitative study about Innu perspectives on end-of-life care
Source: BMC Palliat Care. 2024 May 17;23:121. doi: 10.1186/s12904-024-01431-5 (PMC11100191; doi:10.1186/s12904-024-01431-5)
Supplement: Supplementary file 1 — Supplementary Material 1 [file 12904_2024_1431_MOESM1_ESM.docx]

## Interview Question Guide – Healthcare Professionals

1. What is your experience with delivering palliative (or “end-of-life”) care? What about specifically for the Innu of Sheshatshiu?
2. What resources or supports are available in palliative care delivery for the Innu of Sheshatshiu? This includes community, hospital, and any other context in which palliative care could be provided. What resources are lacking? [Prompt: personnel or staffing, number of beds, space for visitors, interpreters, inexperience, turnover, isolation]
   - Can you describe the usual scenario when a local Sheshatshiu resident presents to a healthcare provider in need of palliative care? [Prompt: How is the need for palliative care identified? Who then provides which types of care, and how are they consulted or otherwise recruited to provide care?]
   - Are there any services, resources, or supports available specifically for Innu patients who receive palliative care in a hospital or clinic setting?
   - What services, resources, or supports are there for patients wishing to remain in their community of Sheshatshiu and receive palliative care there?
   - What supports are available for people in Sheshatshiu who want to die at home?
3. How would you describe the relationship between healthcare delivery and an Innu patient’s culture?
   - How have you found Innu culture and traditions to be an asset or source of strength for a patient requiring palliative care? [Prompt: How have Innu beliefs or practices resulted in the improved delivery of palliative care services? How has their culture improved their quality of life as they approach the end of their life?]
   - What challenges have you found a patient’s culture can present to delivering palliative care to the Innu?
4. Did you receive cultural training before you started working at the [hospital OR clinic] here? *If yes*, what did that look like? What kinds of things did you learn?
   - Did you complete specific training for cultural safety in *palliative* care? If so, what did this look like?
   - What does it mean to you to provide “culturally safe” palliative care?
   - How confident do you feel in your ability to provide culturally safe palliative care to the Innu population of Sheshatshiu?
   - Can you think of any additional supports, resources, or training that would help you provide culturally safe palliative care to the Innu of Sheshatshiu? What are they?
5. What conversations do you have about death and dying with your patients? [Prompt: This means your patients in general, i.e., any or all patients you encounter who need to discuss death and dying.]
   - Do these conversations differ when you’re talking to Innu patients? If yes, in what ways?
   - How much have your Innu patients or their family members told you about what is important to them at the end of their life? What do they say? [Prompt: What are their values? What is important to them? What are their goals of care?] What are the elements of communication? [Prompt: language, silence, body language, eye contact] Are there any barriers to communication?
   - In your experience providing palliative care to Innu patients, who is present at the bedside during the final stages of death? Who is involved in the patient’s care? [Prompt: Can you describe their respective roles?]
6. To what extent is the community involved in the end-of-life process of an Innu patient? Does this differ in hospital and home-based settings?
7. To what extent do you feel that current palliative care delivery meets the needs of the population of Sheshatshiu?
   - Can you describe the challenges to providing culturally safe palliative care to the population of Sheshatshiu?
8. What are some of the ways you have adapted (or seen others adapt) the usual delivery of palliative care to accommodate or respond to the cultural values of the Innu?
   - Can you describe any changes you think should be made to improve the cultural safety of palliative care delivery for the Innu of Sheshatshiu? [Prompt: This could include healthcare system-level changes, or changes in the delivery of patient care, etc.]
