# Supplementary material for: Patshitinikutau Natukunisha Tshishennuat Uitshuau (a place for Elders to spend their last days in life): a qualitative study about Innu perspectives on end-of-life care
Source: BMC Palliat Care. 2024 May 17;23:121. doi: 10.1186/s12904-024-01431-5 (PMC11100191; doi:10.1186/s12904-024-01431-5)
Supplement: Supplementary file 2 — Supplementary Material 2 [file 12904_2024_1431_MOESM2_ESM.docx]

## Interview Question Guide – Decision-Makers

1. Can you describe the delivery of palliative or “end-of-life” care for the Innu from Sheshatshiu? What services, supports, resources are currently available that you are aware of?
   - For institutional-based palliative care?
   - For home-based palliative care?
2. Can you describe any challenges to providing good palliative care in Sheshatshiu?
3. How well are current healthcare policies aligned with culturally safe palliative care? Are you aware of any challenges that have come up in implementing those policies within the healthcare system?
4. How much input do the Innu have in health programs, healthcare policy, and healthcare delivery? [Prompt: What about for palliative care, specifically?]
   - What types of input from the Innu would be helpful to healthcare policy makers? What information would be useful? What do you think the best way to communicate that information would be? [This question is for Provincial Government of NL and Regional Health Authority decision makers.]
   - What do you feel policy makers need to know to develop healthcare policies that address the needs of the Innu? What about end-of-life care, specifically? What do you feel would be the most effective way of sharing that information? [This question is for decision makers from the Innu community.]
5. How sustainable is the current model of palliative care for the Innu of Sheshatshiu? How do the sustainability of hospital-based and home-based palliative care models compare?
   - Are there any specific changes you think should be made to the policies or practices of palliative care for the Innu of Sheshatshiu?
   - Can you advise us how you think these or other such changes should be made in a way that is sustainable for this healthcare system?
6. What is most important to your institution [i.e., Gov of NL, SIFN, LGH, etc.] in creating, maintaining, or revising healthcare services for palliative care for the Innu of Sheshatshiu?
   - If there were any ways in which our project found such services could be improved, how would you like to hear about it? [Prompt: What kind of dissemination materials? A full report, a one-pager, a presentation, etc.] What type of information would be relevant to you in your context?
