# Supplementary material for: Patshitinikutau Natukunisha Tshishennuat Uitshuau (a place for Elders to spend their last days in life): a qualitative study about Innu perspectives on end-of-life care
Source: BMC Palliat Care. 2024 May 17;23:121. doi: 10.1186/s12904-024-01431-5 (PMC11100191; doi:10.1186/s12904-024-01431-5)
Supplement: Supplementary file 3 — Supplementary Material 3 [file 12904_2024_1431_MOESM3_ESM.docx]

## Focus Group Question Guide – Community Members

1. Can you please share your experience of the passing of a loved one?

Follow-up questions (if not already addressed):

- Communication:
  - What information about their illness did you get? Who was there when you were given the information? Who did you talk with?
  - Who did you have contact with during this time (e.g., doctors, nurses, etc.)? How were they (e.g., the doctor, nurses, etc.)?
  - Can you describe their relationship with your family member for me? [Prompt: how much trust was there? How was the communication?]
- If they died in or spent time in a hospital prior to passing:
  - What was your experience like in the hospital setting?
  - How were the room and services? What services did you receive while caring for your loved one?
- If they died in community:
  - Who supported you and your family member through that process?
  - What healthcare professionals were involved?
  - What did they do?
- Assessment:
  - What things were done well?
  - What things need improvement?

1. What is your vision of the way an Innu person would ideally continue on their journey from this life to the spirit world?

Follow-up questions (if not already addressed):

- In your opinion, what does a peaceful death look like? [Prompt: at home or in the hospital?]
- Who should be involved in the patient’s care? Who should be present during the final stages of dying?
- What practices are important for the Innu to do during the final hours of life? [Prompt: conversation, song, prayer?]
- How do others take part in this?
  - Friends and family
  - Other community members
- Where do you think you would want to die if you were ill?

1. How well do your beliefs about end-of-life care match the medical community’s approach to end-of-life care?

Follow-up questions (if not already addressed):

- What are the good things you have seen healthcare professionals do to take care of dying patients from Sheshatshiu?
- What changes would improve the way end-of-life care is delivered to better meet the cultural and spiritual needs of the Innu?

1. Is there anything else you would like to add to this interview?
